# Enhancing Human Health and Wellbeing through Sustainably and Equitably Unlocking a Healthy Ocean’s Potential

**DOI:** 10.5334/aogh.4471

**Published:** 2024-07-09

**Authors:** Lora E. Fleming, Philip J. Landrigan, Oliver S. Ashford, Ella M. Whitman, Amy Swift, William H. Gerwick, Johanna J. Heymans, Christina C. Hicks, Karyn Morrissey, Mathew P. White, Lota Alcantara-Creencia, Karen A. Alexander, Thomas Astell-Burt, Roberto G. S. Berlinck, Philippa J. Cohen, Richard Hixson, Mohammad Mahmudul Islam, Arihiro Iwasaki, Radisti A. Praptiwi, Hervé Raps, Jan Yves Remy, Georgina Sowman, Eva Ternon, Torsten Thiele, Shakuntala H. Thilsted, Jacqueline Uku, Stephanie Ockenden, Pushpam Kumar

**Affiliations:** 1 European Centre for Environment and Human Health of the University of Exeter Medical School, Cornwall, UK; 2 Instituto de Quimica de Sao Carlos, Universidade de Sao Paulo, Sao Carlos, SP, Brazil; 3 Ocean Program, at World Resources Institute, London, UK; 4 Program for Global Public Health and the Common Good, Boston College, Boston, Massachusetts, USA and the Centre Scientifique de Monaco, Monaco; 5 Skaggs School of Pharmacy and Pharmaceutical Sciences, University of California San Diego, San Diego, US; 6 European Marine Board, Ostende, Belgium; 7 Environment Centre, Lancaster University, Lancaster, UK; 8 Department of Technology, Management and Economics, Technical University of Denmark, Denmark; 9 University of Vienna, Austria, Vienna; 10 College of Fisheries and Aquatic Sciences, Western Philippines University, Palawan, Philippines; 11 Marine Governance and Blue Economy at Heriot-Watt University, Orkney, UK; 12 School of Architecture, Design and Planning, University of Sydney, Sydney, Australia; 13 Small-Scale Fisheries Research Program World Fish, Penang, Malaysia; 14 Critical Care, County Durham and Darlington NHS Foundation Trust, Darlington, UK; 15 Department of Coastal and Marine Fisheries, Sylhet Agricultural University, Sylhet, Bangladesh; 16 Department of Applied Chemistry, Faculty of Science and Engineering, Chuo University, Tokyo, Japan; 17 Research Center for Ecology and Ethnobiology, National Research and Innovation Agency (BRIN), Jakarta, Indonesia; 18 Centre Scientifique de Monaco, Monaco; 19 Shridath Ramphal Centre, the University of the West Indies, St. Lucia/Barbados; 20 Advanced Wellbeing Research Centre, Sheffield Hallam University, Sheffield, UK; 21 Laboratoire d’Océanographie de Villefranche at Sorbonne Université, Paris, France; 22 Research Institute for Sustainability – Helmholtz Centre Potsdam (RIFS), Potsdam, Germany; 23 Nutrition, Health and Food Security Impact Area Platform Worldfish CGIAR, Penang, Malaysia; 24 Kenya Marine and Fisheries Research Institute, Mombasa, Kenya; 25 UNEP, Washington, DC, US

**Keywords:** biodiversity, equity, environmental justice, marine protected areas (MPAs), biotechnology, natural products, seafood, blue economy, blue health

## Abstract

A healthy ocean is essential for human health, and yet the links between the ocean and human health are often overlooked. By providing new medicines, technologies, energy, foods, recreation, and inspiration, the ocean has the potential to enhance human health and wellbeing. However, climate change, pollution, biodiversity loss, and inequity threaten both ocean and human health. Sustainable realisation of the ocean’s health benefits will require overcoming these challenges through equitable partnerships, enforcement of laws and treaties, robust monitoring, and use of metrics that assess both the ocean’s natural capital and human wellbeing. Achieving this will require an explicit focus on human rights, equity, sustainability, and social justice. In addition to highlighting the potential unique role of the healthcare sector, we offer science-based recommendations to protect both ocean health and human health, and we highlight the unique potential of the healthcare sector tolead this effort.

## Introduction

The health and wellbeing of all people depend on the health of the ocean (see Table 1). Photosynthetic microorganisms in the sea generate 50% of the oxygen produced each year and 80% of all oxygen ever generated [1]. By absorbing 25% of all CO_2_ emissions and more than 90% of excess atmospheric heat [2,3], the ocean stabilises the climate, slows global warming, and enables the thriving of civilisations.

**Table 1:** Selected direct and indirect risks, benefits, and opportunities for human health and wellbeing from interactions with the ocean.

BENEFIT/OPPORTUNITY	HEALTHY OCEAN BENEFIT	POTENTIAL HUMAN HEALTH AND WELLBEING BENEFIT	CITATIONS
**Climate and weather**	The ocean is critical to the fight against climate change.	Prevention of injury, death, and mental health impacts from extreme weather	Villasante et al. 2023 [3]; Falkenberg et al. 2023 [4]
**Heat and CO**_2_ **sink**	The ocean absorbs 25% of all CO_2_ emissions and more than 90% of excess atmospheric heat.	Prevention of extreme heat, crop loss, starvation	Hoegh-Guldberg et al. 2023 [2]
**Oxygen**	The ocean sustains all life on earth by providing 50% of the oxygen produced on earth each year and 80% of all the oxygen ever created.	Prevention of crop and other biodiversity loss	Grégoire et al. 2023 [1]
**Biodiversity (including marine protected areas [MPAs])**	Emerging research with communities living in/around MPAs and other areas designated as protected; diverse human health and wellbeing benefits; and collaborative and effective management with ongoing involvement of local communities is essential toward creating and sustaining these ocean and human health benefits.	Livelihoods, improved nutrition, decreased overall national mortality, and improved child health as well as positive ecosystem impacts	Winther et al. 2020 [5]; Madarcos et al. 2021 [6]; Haque et al. 2023 [7]; Nowakowski et al. 2023 [8]; Ban et al. 2019 [9]; Gollan and Barclay 2020 [10]; Rasheed 2020 [11]
**Livelihoods and** **economics**	The ocean is a source of wealth. The ocean economy is estimated to generate US $1.5–2.5 trillion annually and to provide jobs for more than 30 million people.	Seafood as nutrition and prevention of NCDs and mental health impacts	OECD 2016 [12]; Ocean Panel 2020 [13]
**Marine Biotechnology (including marine drugs)**	Thirty thousand unique molecules and 10% of currently known natural products have been discovered in marine life; 23 approved pharmaceutical agents have been developed from marine molecules, and an additional 33 are in clinical trials. The ocean is a source of new medicines and biotechnologies, from essential pain medicines to plastic alternatives to essential DNA libraries.	Development of treatments for inflammation, immune system disorders, skin pathologies, infectious diseases, NCDs, and cancers Alternatives to plastics and creation of sustainable other biomaterials	Antunes et al. 2023 [14]; Bouley et al. 2023 [15]; CHEMnetBASE 2023 [16]; Pascual Alonso et al. 2023 [17]
**Seafood and** **food** **security**	For more than 3 billion people, nearly 40% of the world’s population, the ocean is an essential source of food and livelihood.	Prevention of starvation, childhood stunting, NCDs	FAO Duke University & WorldFish 2022 [18]; Maycock et al. 2023 [19] Golden, et al. 2021 [17]; Tigchelaar et al. 2022 [20]; Naylor et al. 2021 [21]; Golden et al. 2016 [22]
**Blue** **spaces (including culture)**	Interactions with the ocean and with other blue spaces enhance the physical health and mental wellbeing of humans from infancy to old age.	Support culture, physical health, and mental wellbeing	White et al. 2020 [23]; Fleming et al. 2019 [24]
**Threat**	**Unhealthy Ocean Risks**	**Potential Human Health and Wellbeing Risks**	**Citations**
**Heat**	As the ocean absorbs more heat, the sea surface temperature rises: increased frequency of extreme weather, polar ice melting, sea level rise, and coastal flooding; migration of fish stocks from dependent communities; increased harmful algal blooms (HABs); and pathogen spread.	Death Injury Infectious diseases Starvation HAB illnesses Mental health NCDs Disrupt cultural integrity	Nash et al. 2017 [25]; Falkenberg et al. 2023 [4]
**Acid**	Increased atmospheric CO_2_ is absorbed by the ocean; low pH dissolves coral, shellfish, and calcium-containing microorganisms that sustain the entire marine web, and impacts fisheries.	Starvation Obesity Mental health NCDs Disrupt cultural integrity	Nash et al. 2017 [25]; Falkenberg et al. 2023 [4]
**Deoxygenation**	Dissolved ocean oxygen decreases as oceans become warmer and more acidic: oceanic ‘dead zones’ impact fisheries.	Starvation Obesity Mental health NCDs Disrupt cultural integrity	Grégoire et al. 2023 [1]; Falkenberg et al. 2020 [26]
**Overfishing**	Destructive industrial fishing practices, with rising temperatures and pollution, damage ocean ecosystems and biodiversity, and deplete fisheries.	Starvation Obesity Mental health NCDs Disrupt cultural integrity	FAO Duke University & WorldFish 2022 [18]; Maycock et al. 2023 [19]; Golden, et al. 2021 [17]; Tigchelaar et al. 2022 [20]; Naylor et al. 2021 [21]; Golden et al. 2016 [22]
**Oil and gas extraction**	Fossil fuel extraction and transport release toxic hydrocarbons, increasing climate change; oil spills injure and kill marine organisms, destroy biodiversity, and impact fisheries.	Death Injury Mental health Increased NCDs Disrupt cultural integrity	Nash et al. 2017 [25]; Landrigan et al. 2020 [27]; Lelieveld et al. 2019 [28]
**Deep-sea mining**	Deep-sea mining may damages the seabed and vulnerable habitats, releases radiation, and impacts fisheries.	Obesity NCDs Cancer Starvation Disrupt cultural integrity	Landrigan et al. 2020; [27] Hamley 2022 [29]; Miller et al. 2021 [30]
**Pollution**	Eighty percent of pollution arises on land from human activities: plastics, heavy metals, petroleum waste, manufactured chemicals, pesticides, radiation, and nutrients (including sewage); these pollutants damage ecological health and biodiversity, and impact fisheries.	HAB illnesses Neurotoxicity Foetal/developmental toxicity Reproductive toxicity Mental health NCDs Cancer Disrupt cultural integrity	Landrigan et al. 2020 [25]; Landrigan et al. 2023 [27]; Short et al. 2021 [31]
**Economics**	Profit-driven, ocean-based economic development offers short-term economic gain, with no concern for ocean health, the health and wellbeing of marginalised coastal communities, biodiversity, and marine degradation.	Occupational injury and death Starvation Mental health Disrupt cultural integrity	Germond-Duret et al. 2022; Das 2023 [32,33]

The ocean is a source of great wealth. More than one-third (2.75 billion) of the world’s population lives within 100 km from the coast and 14 of the world’s 17 megacities are located on the coasts [34]. The ocean economy generates more than US $1.5–2.5 trillion per year, provides over 30 million formal jobs [12], and supports the livelihoods of another 500 million people employed informally in artisanal and small-scale fisheries [18].

The ocean is a major source of food, preventing malnutrition and potentially chronic diseases. Fish and other aquatic foods feed over three billion people, 40% of the world’s population [35,19].

The ocean provides joy, peace, recreation, and spiritual sustenance. For thousands of years, it has inspired art and shaped cultures. Interactions with blue spaces (coasts, salt marshes, beaches, and the ocean itself) promote physical health, mental health, and social wellbeing from infancy to old age and have been shown to extend longevity [23,24]. For all these reasons, the coasts and ocean have become major destinations for tourism, wellbeing interventions, and retirement [36].

Unique molecules and organisms found only in marine species have provided multiple essential medicines and some of the world’s strongest adhesives [37]. The extraordinary structures of marine organisms have inspired breakthroughs in architecture and engineering [14,15].

Properly managed, the resources of the ocean have the potential to sustainably and equitably benefit humanity through increased employment, an enhanced economy, and strengthened infrastructure (including healthcare), while reducing environmental risks, ecological scarcities, hunger, and social injustices [38,5,13,39].

**Figure 1:**
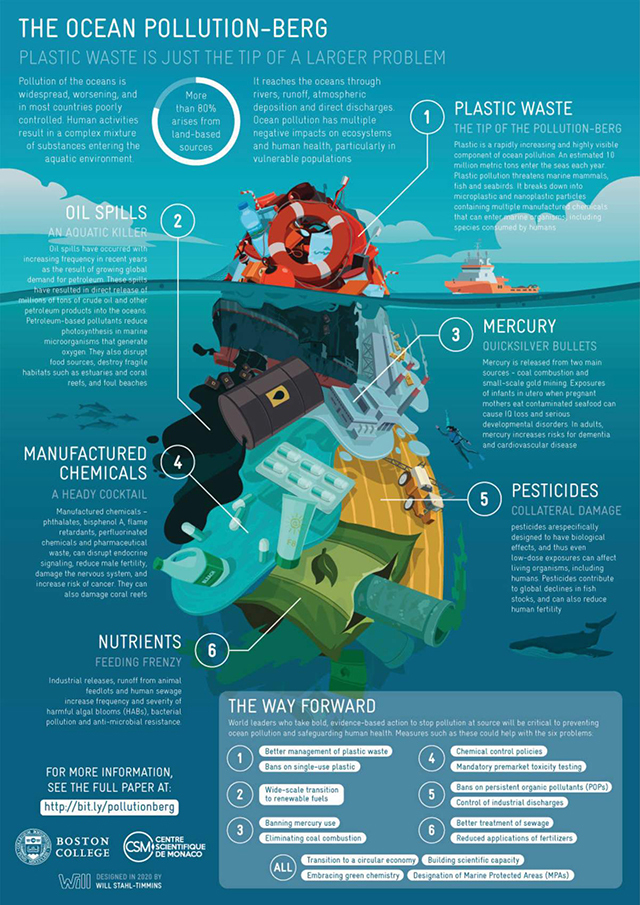
The Ocean Pollution-Berg: Plastic waste is just the tip of a larger ocean pollution problem (source: artist Will Stahl-Timmins; Landrigan et al. 2020 [27]).

However, the ocean is under threat, and current threats to the health of the ocean impede the realisation of its potential benefits [40]. These threats are largely of human origin (Table 1). They include the worsening impacts of climate change (e.g., sea surface warming, violent storms, sea level rise, coastal flooding, ocean acidification); pollution by plastics, petrochemicals, and heavy metals from industry and agriculture (see **Figure 1**); unchecked coastal urbanisation that releases nutrients, pharmaceuticals, and microbial pathogens into the inshore ocean; oil and gas extraction; deep-sea mining; loss of biological diversity; and illegal, industrial, and distant-water fishing (see Table 1 for selected references).

The consequences of these diverse and complex threats are increasing damage to marine ecosystems, especially in sensitive areas such as coral reefs, and to the depletion of fisheries [25,27,26,41]. Ultimately these pressures could lead to declines in the biomass of marine photosynthetic species, thus reducing the ocean’s capacity to store CO_2_ and generate oxygen [42,43].

All of these threats to ocean health can directly and indirectly cause harm to human health and wellbeing by increasing risks of injury, malnutrition, gastrointestinal illnesses and other communicable illnesses, mental health problems, and non-communicable diseases (NCDs) such as cardiovascular disease and cancer (see Table 1) [24,27,44]. Because the threats to ocean health are largely of human origin, they are preventable.

**Figure 2:**
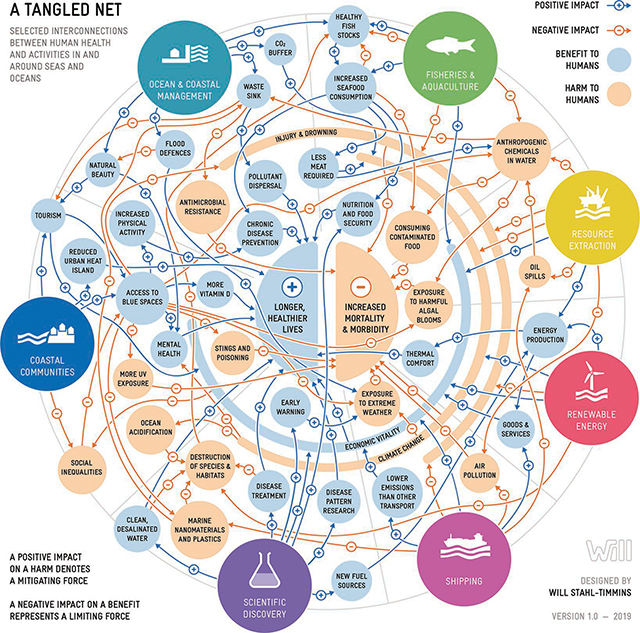
A tangled net: complexity of selected interconnections between human health and activities in and around seas and oceans (Source: artist Will Stahl Timmins; Fleming et al. 2019 [24]).

Recognition is growing that the current threats to the ocean constitute a global emergency, and that urgent global action is needed to protect ocean health and thereby safeguard the health of all humanity. Conversations about the relationships between the ocean and human health have typically focused on risks and threats. They are part of growing discussions around humanity’s negative impacts on planetary health [45]. But that is only one side of the story (**Figure 2**). The ocean offers many opportunities for improving human health and wellbeing, and the vast magnitude of these opportunities is only beginning to be appreciated (see Table 1) [5, 46].

In this viewpoint, we explore the emerging opportunities that the ocean holds for humanity through a brief overview of the current evidence and the presentation of brief case studies (see Box 1 **Definitions**). And second, we offer regional, national, and international policymakers as well as all citizens of the world a selection of achievable actions for equitably and sustainably improving human health and wellbeing through realisation of the ocean’s resources. We examine these opportunities in four key areas:

- Medicine and biotechnology
- Sustainable food security
- Physical, mental, and social health and wellbeing
- The economy and equity

**Box 1. Definitions**

We use ‘ocean’ and ‘oceans’ interchangeably; and we define the seas, coasts, and ocean as ‘healthy’ when they are resilient, productive, and diverse [47].

The United Nations (UN) Brundtland Commission (1987) defined ‘sustainability’ as ‘meeting the needs of the present without compromising the ability of future generations to meet their own needs’; and the UN has defined ‘equity ’ as ‘fair and equal distribution of power, economic resources, opportunities, goods and services across the social spectrum’.

Human ‘health’ is defined as ‘a state of complete physical, mental and social wellbeing and not merely the absence of disease or infirmity’ [48]. We use ‘wellbeing’ as a positive state experienced by individuals and societies. Wellbeing encompasses both physical health and mental health, and is determined by social, racial economic, environmental, and historical conditions [49].

We conclude by advancing the argument that if new medicines, technologies, and foods are to continue to come from the ocean, and if the ocean is to continue to provide health, wellbeing, livelihoods, recreation, and inspiration to people around the world, we must work together to preserve and protect its resources. We highlight the potential role of the healthcare sector to advocate for and actualise more sustainable interactions between the ocean and human health. And regardless, as we realise the ocean’s great benefits, we must ensure that these benefits are realised sustainably and equitably [5,40,50].

## Ocean-Based Opportunities for Humanity

### Medicine and biotechnology

The rich biodiversity of the ocean holds many opportunities for promoting and enhancing human health and wellbeing. The ocean has the potential to be a sustainable source of new medicines and biotechnologies, ranging from pain medicines and cancer drugs to plastic alternatives and new chemical catalysts [14,15].

To date, 23 pharmaceutical agents have been developed and approved from marine molecules, and an additional 33 are in clinical trials and development [14]. These therapeutics have been used already for treatment of cancers, infectious diseases, immune system disorders, skin pathologies, and inflammation [16,17] (see **Case Study 1**).

**Case Study 1. Anticancer medicines from marine cyanobacteria**

The Cyanobacteria (blue-green algae) are an ancient group of organisms that arose on earth over two billion years ago. They are abundant producers of biologically active substances. Larger marine organisms that feed on cyanobacteria, such as sea slugs, can accumulate these biologically active compounds and use them in their own defense against predators [14].

Several cyanobacterial compounds extracted from sea slugs show great promise for the treatment of diseases such as cancer. For example, dolastatin 10 is a natural product originally discovered in an Indian Ocean sea slug, *Dolabella auricularia*, which has extremely potent antitumour activity, and related medications have been developed to successfully treat cancer [51].

**Figure 3:**
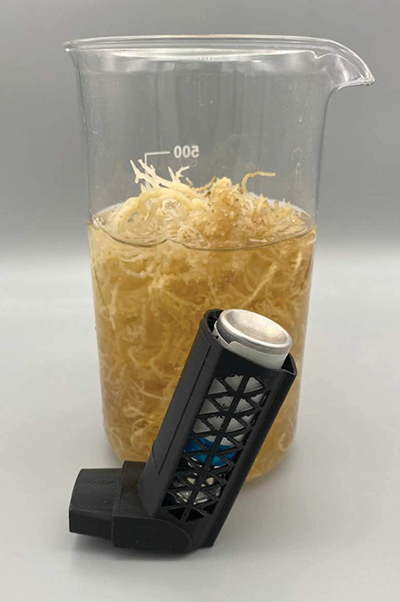
Seaweed-based biomaterial used to 3D print inhaler prototype (front), set against a jar of the seaweed, used as an alternative feedstock to fossil fuels used in current medical device manufacturing (back). Source: with permission: Symbio-tex (https://www.symbio-tex.com/).

Products such as food supplements, fuels, and nanoparticles manufactured from marine resources have the potential to generate less waste and less CO_2_ than current manufacturing processes, which are based largely on fossil carbon [52,53]. For example, farmed seaweed commands a high value for food, cosmetic, and medical purposes (Figure 3). Seaweeds are rich also in essential nutrients (carotenoids, vitamins, and phenolic antioxidants), and thus may help to mitigate the nutrient-poor diets of many coastal populations; these materials can be produced by socially conscious and environmentally and economically sustainable aquacultural methods, including large-scale production [54].

An additional benefit of seaweed cultivation is that it can also sustain non-chemical agriculture on land and in the ocean; for example, metabolites contained in seaweed can stimulate crop growth to improve agricultural food production on land without the addition of fossil fuel–based nutrients [55]. By significantly reducing methane emissions from livestock farming, red seaweeds are currently being explored as a supplement for dairy and beef cows; methane from ruminant animals is responsible for approximately 15% of the global anthropogenic greenhouse gas emissions driving global climate change [2,56].

Most marine biodiversity is still undocumented, and marine organisms’ unique adaptative traits are still largely undiscovered. Only 3,300 of the 1.5 million known animal species on earth have had the DNA of their genomes fully sequenced [57]. This rich and still largely unexplored biological diversity offers important potential benefits for human health and wellbeing in terms of food, medicines, and marine biotechnology, as well as opportunities for sustainably and equitably strengthening the global economy [40]. Properly accessed, these benefits could address many of the challenges that surround us in the current age and sustain humanity in the future [14,15].

To protect and explore this biodiversity, there is an urgent need to undertake periodic inventories of marine organisms; conduct detailed biochemical investigations of adaptive traits; and initiate studies of accelerated evolution, potentially through ethical public–private partnerships. Open access to these data is essential to provide equitable, sustainable, and creative development and use by all, not ownership and use by the few [58].

### Sustainable food security

Fish and other aquatic foods feed more than three billion people, nearly 40% of the world’s population [35]. Properly managed, the ocean could produce enough food to significantly nourish the entire population of the globe [18,19,59,20].

The production of food from the sea is a major source of employment and income. Wild fisheries, aquaculture operations, and the fishery supply chain support the work of more than 500 million people worldwide, most engaged in small-scale fisheries in low and middle-income countries (LMICs). They provide livelihood and food security for communities in every country, including for women and indigenous communities [18,59,20].

In rural and remote areas in low- and middle-income countries where land-based diets may be lacking in key micronutrients, locally available fish and other seafood harvested from healthy ocean ecosystems can provide vital nutrition and micronutrients, preventing malnutrition particularly in children [60,61]. For example, seafood products are increasingly used as key ingredients in emergency supplementary and ready-to-use therapeutic food programmes [62,63], as well as in school feeding programmes [64], where they play a critical role in preventing micronutrient deficiency and its health consequences [65] (see **Case Study 2**). Seafood may become an increasingly important source of nutrition in such areas in future years, because increased concentrations of atmospheric carbon dioxide from climate change appear to reduce levels of protein, zinc, and iron in staple land-based food crops [66].

Ongoing efforts to scale up the use of local aquatic foods in feeding programmes can address immediate nutritional needs, offer long-lasting coping strategies, and generate local income (**Case Study 2**). These programmes are most effective when the food is procured locally and local knowledge is integrated with the latest nutritional insights to enhance the potential benefits [65].

**Case Study 2. Inclusion of small fish powder in the meals of children in Anganwadis and Primary Schools, in Assam, India**

*Matsya Paripushti* (Complete Nourishment through Fish) is a newly initiated feeding programme by the Assam state government that includes the addition of a powder made from small fish to the midday meals of 4,000 children aged 3–6 years attending rural health care centers and children aged 6–10 years in lower primary schools in the Kamrup district. The weight of all children under five years of age and body mass index in older children are being monitored and will be used to compare children consuming small fish powder with those who are not. The goal is to improve the nutrition and health of children, through improving dietary diversity and increasing the intake of micronutrients, essential fatty acids, and protein [67].

Current challenges to the health of the ocean threaten food security and increase the risk of malnutrition (both from starvation and obesity), particularly for children and coastal indigenous populations [19]. These challenges include climate change, pollution, and loss of marine biodiversity; improper ocean governance; ineffective, inequitable, and unsustainable fisheries management; poverty; and the inequitable distribution of seafood [18,5,68,69].

### Physical, mental, and social health and wellbeing

A growing body of evidence demonstrates that spending time in, on, and by the ocean enhances physical health and mental wellbeing and that interacting with a healthy ocean can contribute to ‘the fundamental right of every human being…to enjoy… the highest attainable standard of health’: [70].

The benefits of residence near the coast are particularly strong among poorer communities with high levels of environmental and socio-economic disadvantage [71] and during times of great stress such as financial downturns and the COVID-19 pandemic [72]. The disproportionate benefits of coastal residence are seen globally and are by no means restricted to the Global North (see **Case Study 3**) [73,74,75].

**Case Study 3: Thriving during the COVID-19 Pandemic (Indonesia): The ocean as a source of solace in times of stress**

Artisanal fishing is the predominant work of small island communities in Indonesia, and these communities rely on local coastal and marine ecosystems for their food and their economic livelihoods. Emerging evidence indicates that ecosystems in small island communities in Indonesia are important sources not only of sustenance, but also of health and wellbeing.

Lockdown measures imposed during the COVID-19 pandemic resulted in economic loss, increased household conflict, and diminished access to healthcare in these often remote communities. However, engagement in ocean-based recreational activities during this challenging time, especially collective immersive interactions such as swimming and snorkeling, were shown to protect, or ‘buffer’, people against adverse mental health outcomes [73,74].

The benefits of engaging in marine recreational activities for mental and social wellbeing are not restricted to affluent post-industrial societies in the Global North.

The Global Burden of Disease study [76] finds that the disease burdens in many countries are shifting from communicable, nutritional, and neonatal issues to non-communicable diseases (NCDs) such as cardiovascular diseases, diabetes, depression, and cancer as these countries develop economically, urbanise, and industrialise. Strategies to prevent NCDs that are developed in individuals and communities living near the ocean may offer important and widely generalisable health benefits (See **Case Studies 3** and **4**) [44].

**Case Study 4: Blue health and wellbeing ‘prescriptions’: The Bay (Morecombe, UK)**

Morecombe Bay is a large stretch of coastline in North West England (UK). Working closely with local wildlife trusts and the Eden Project, the Lancashire and South Cumbria National Health Service (NHS) Foundation Trust has established ‘The Bay’, an ocean-based wellbeing programme along the whole of the bay’s coastline.

Focusing on ‘blue social prescribing,’ healthcare providers refer patients to programmes organised by and supporting different local coastal communities. The Bay Programme offers a range of interventions that encourage greater coastal use to both treat and prevent chronic health conditions. Nearly 500 individuals will be supported over the first two years of the programme’s delivery.

The social, environmental, and economic benefits of blue social prescribing are extensive. Early social return on investment (SROI) analyses suggest that for every £1 invested in Bay activities, there is £2.16 of benefit in terms of reduced costs of treating mental health-related conditions [77]. This return is conservative, as it does not consider the wider economic benefits of reducing unemployment, increasing visitors to the coastline, and environmental cost savings from the positive work achieved.

Marine protected areas (MPAs) offer particular benefits for physical and mental health. Among communities in the Global South living in and near MPAs, emerging research demonstrates that residence in these areas provides multiple benefits to human health and wellbeing, including decreased all-cause mortality, improved child health, and extended longevity. Positive ecosystem impacts are also seen (see **Case Study 3**) [6,7,8].

Resolutions from the UN Human Rights Council in 2021 (A/HRC/RES/48/13), and the UN General Assembly in 2022 (A/RES/76/300) recognise that a clean, healthy, and sustainable environment is a human right. In keeping with these resolutions, governments must take great care when they designate coastal and island ecosystems as MPAs to recognise and respect the rights of the people who live in and near these areas. Specifically, they must seek to minimise the negative impacts of MPA designation on population displacement, erosion of traditional marine tenure, loss of livelihoods, geographic exclusion, and inequitable benefit sharing (c.f. [78]).

These goals can be achieved through meaningful engagement with local communities, abiding by customary norms and institutions, and respect for traditional, area-based conservation measures (OECMs). Such participatory, community-based approaches are essential to ensure the sustainable future of both people and the ​​ocean [79,9,10,11].

### The economy and equity

The ocean is a source of enormous wealth and is estimated to generate US $1.5–2.5 trillion annually [12]. The ocean provides formal jobs for more than 30 million people, including workers engaged in formal fishing operations, mariners, and workers in the global coastal tourism and recreation industries [12]. Additionally, it supports the livelihoods of millions more people informally employed in artisanal and small-scale fisheries [18].

Properly managed, ocean resources have the potential to benefit all sectors of society in all countries through increased employment opportunities, enhanced economic revenues, strengthened infrastructure, and human health and wellbeing, while reducing environmental risks, biodiversity loss and ecological scarcities, and social injustices [38,5].

Contravening that egalitarian vision, extractive ocean industries such as bottom dredging, distant-water fisheries, and oil drilling, which often operate under the banner of the ‘Blue Economy’, are degrading the ocean environment, releasing vast quantities of greenhouse gases, polluting air and water, depleting marine resources, and deepening global economic inequity [39,80]. These industries are guided by a linear economic paradigm that focuses single-mindedly on short-term gain as measured by the Gross Domestic Product (GDP), views natural resources and human capital as abundant and expendable, and gives no heed to the consequences of their reckless exploitation. This paradigm fails to link economic gain to social justice or to maintenance of the earth’s resources. It is not sustainable [13,81].

To deliver sustainable health and wellbeing outcomes for all, the ocean economy must position equity at the core of its agenda. It must encourage economic development without ecosystem destruction and prize sustainability over short-term profit taking [13,39].

Key to measuring progress toward a more sustainable economy will be the deployment and routine use of economic metrics that look beyond GDP and short-term gain and quantify the ‘inclusive wealth’ of nations. Inclusive wealth is defined as the aggregate value of all capital assets, where the value of a unit of a capital asset is measured by the contribution it makes to increasing current and future human wellbeing [81,82]. A nation’s inclusive wealth includes the value of its natural resources (natural capital) and its human resources (human capital).

Measures of natural capital quantify the full benefits of nature and allow assessment of the costs of natural resource depletion. Routine use of these measures thus has potential to expand cost-benefit assessments and widen decision-making beyond a narrow focus on short-term profit [81]. An example is the calculation of the economic value of the flood control ecosystem services provided by wetlands, salt marshes, and mangrove forests compared to the costs of destroying these natural coastal systems and replacing them with concrete flood barriers and beachfront hotels [83].

To measure progress toward a more sustainable ocean economy, a framework is required that assesses blue natural capital. The urgency of deploying such a framework is underscored by the findings of

The latest Inclusive Wealth Report from the UN Environment Programme (UNEP), which finds that the natural capital of 163 countries declined in the 27-year period from 1992 to 2019 [81]. Proper quantification and valuation of blue natural capital can slow the depletion of marine resources. It can help integrate consideration of ocean resources, including blue carbon, into conventional decision-making and counterbalance shortsighted economic drivers [81].

A promising strategy for encouraging inclusive growth that improves the health and wellbeing of coastal communities is ‘social innovation’. Social innovation is defined as a process whereby relevant actors and institutions join in self-organising, community-driven networks to develop new and improved ways of collaborative action [84]. The core objective is to deliver bottom-up behavioural change across multiple actors [85].

A positive example of such intervention is seen in the case of small-scale fisheries in LMICs, where prioritisation of equity and human health has been shown to increase ocean health, improve nutrition, enhance food security, stabilise local economies, and increase physical and mental wellbeing across all age groups (see **Case Study 5**) [84,86,87,88].

**Case Study 5. Bangladeshi fishers’ collective action to create inclusive growth for ocean and human health**

The hilsa shad (*Tenualosa ilisha*) fishery is Bangladesh’s single most important fishery, employing 50,000 people, predominantly artisanal fishers.

Access to fishing equipment and to lucrative urban markets has traditionally been largely controlled by middlemen, presenting a structural problem in the hilsa industry. Fish brokers secure their hold on the industry by extending investment capital to fishers at high interest rates, thus locking fishers into poverty, jeopardising their economic security, and threatening their health and wellbeing. The hilsa fishers’ precarious existence is further endangered by increasing numbers of climate change–driven tropical depressions and cyclones in the Bay of Bengal.

To counter these negative economic pressures, fishers in several villages along the southern coast of Bangladesh have begun to form rural self-developing societies/cooperatives that extend financial support and equipment to their members. These groups hold potential for protecting community interests, enhancing communal and individual health and wellbeing, and promoting ocean stewardship.

Within rural fishing communities that have formed cooperatives, reports show that increased social cohesion and collective action are emerging as a basis for future adaptation strategies, while positive support within the group helps their mental health. These cooperatives also benefit physical health, for example, by providing collateral-free loans to support food and nutritional security during seasonal fishery closures and to cover the costs of treatment during illness [87].

Building alternative skills and self-help rural infrastructure development could also support better market access towards enhancing fishery income and poverty alleviation programmes. The challenge remains of how to support such initiatives to maintain their self-determination in the face of powerful and competing interests that are able to deliver, and profit from, economies of scale.

### The particular role of health professionals and the health sector

Because health professionals are trusted members of societies, who are seen and, in fact, are advocates for their patients as well as experts in human health and wellbeing, the health sector is uniquely well positioned to lead the way in advocating for sustained global action to protect ocean health [89,90,91]. Indeed, the health sector has come to recognise that ‘the overall environmental crisis is now so severe as to be a global health emergency’ [50].

However, many health professionals are unaware of or do not have the time and support to engage with the mounting body of literature on environmental threats to human health or the emerging literature indicating that contact with the ocean can improve mental and physical health [91]. They are not aware of the growing evidence which shows that physician-recommended interactions with nature (so-called social prescribing, or, in the case of the ocean, blue prescribing) can reduce risk and slow progression of NCDs while also enhancing mental health [92,93].

Greater engagement of the ocean health community with medical doctors, nurses, and public health professionals (and vice versa) is needed to engage, motivate, support, and secure buy-in from this influential group (see **Case Study 6**) [94,95].

**Case Study 6. The National Health Services: potential for positive action for ocean health**

Healthcare has significant unintentional negative impacts on ocean health, including greenhouse gas emissions, pharmaceutical and plastic pollution, and destructive land use [96].

At the same time, healthcare systems are unique in their ability to support health and wellbeing within communities through place-based approaches that reduce inequalities in coastal and blue spaces at the patient, policy, and population level. For example, connecting patients with nature (including the ocean) through social prescribing programmes (blue prescriptions) can improve physical and psychological health outcomes and potentially restore and protect nature [92,93].

At the policy level, the health sector can play a key role in advocating for improved blue space infrastructure and quality with equitable access; for decreased use of fossil fuels, plastics, and other chemicals; and for biodiverse and clean coastal waters that encourage physical activity and restore mental health (see Healthcare Ocean https://www.healthcareocean.org) [97].

Involving health professionals and the health sector in protecting ocean health will necessitate innovative efforts across multiple areas, including the education of all healthcare professionals, reducing the significant carbon footprint of the health sector [98,99], reducing medical waste and pollution, supporting science-based advocacy on behalf of patients, encouraging healthcare provider involvement with resources and evidence, and placing a greater emphasis on population health and prevention [50,89,91,97]. These efforts will extend into many areas, including energy, transport, supply chains, food, and education. The entire health sector must be involved, including hospitals, healthcare systems, public health, biotechnology, pharmaceuticals, social care, and Indigenous health practices (see **Case Study 7**).

**Case Study 7. Decarbonising healthcare’s maritime logistics**

Emissions from maritime shipping activities for the healthcare sector have significant impacts on planetary and human health. For example, emissions from the UK National Health Service (NHS) supply chain represent 62% of its overall greenhouse gas (GHG) footprint (https://www.england.nhs.uk/greenernhs/a-net-zero-nhs/).

International shipping transports 80% of the medical and other supplies to the UK NHS; shipping is currently a carbon-intensive industry [100]. Therefore, decarbonisation of the maritime shipping sector is essential to achieve the NHS net-zero goals. Changes to reduce associated maritime emissions from the healthcare sector can be amplified across multiple sectors, accelerating positive change, and be taken up by the national health services in all countries globally.

The health sector is also uniquely positioned to model positive change. It can lead the way in encouraging industry leaders across diverse sectors and suppliers to place nature and the ocean at the center of all strategies through understanding, monitoring, and decreasing its own environmental footprint; supporting healthcare personnel and health sector suppliers with resources and evidence; helping ocean recovery; re-examining equitable connections to our coasts; and ensuring that all initiatives benefit both human and all our natural environments [95,96].

## Where We Stand Today and the Path Forward

In this viewpoint, we have argued that the health and wellbeing of humanity, and indeed of all life on earth, are intimately linked to the health of the ocean. We have presented evidence documenting that a healthy ocean benefits human health and wellbeing in myriad ways.

We note, however, that the ocean’s benefits are threatened by climate change, worsening pollution, loss of biodiversity, and deepening economic and social inequalities. All of these threats to ocean health are of human origin. They are all driven by the uncontrolled quest for short-term economic gain (particularly by the Global North, China, and by multinational corporations) without heed for human or environmental consequences and with no concern for long-term sustainability [45,50]. And they can directly and indirectly negatively impact human health and wellbeing.

Key to overcoming these current challenges to ocean health will be cross-sectoral, cross-national partnerships and a global structure of laws, treaties, guidelines, and international organisations [13].

Even though action in protecting ocean health has to date been slow and fragmented, there are an abundance of positive activities at the local, national, regional, and international levels (see Table 2) [101]. On the international scale, these include the following:

**Table 2:** Hypothetical health impacts of international marine laws (source: Carvalho et al. 2023 [101]).

LEGAL FRAMEWORK/CONVENTION	IMPACT
Exclusive Economic Zones (EEZ) under United Nations Convention on the Law of the Sea (UNCLOS)	Equitable distribution of ocean resources and marine protections between nations for energy production, food distribution, and other sovereign uses of the seas
South China Sea (SCS) Arbitration	Suppression of potential conflict between nations that would precipitate a humanitarian crisis for many Pacific and Southeast Asian countries
Convention on International Trade in Endangered Species (CITES)	Protection of endangered species critical to the global food web
Convention on Biological Diversity (CBD)	Conservation of environmental resources and governance of genetic resources for equitable distribution among human populations
Port State Measures Agreement (PSMA)	Decreasing illegal, unreported, and unregulated fishing of stocks necessary for the subsistence of multiple human populations
International Whaling Commission (IWC)	Regulation of whaling for native populations while protecting endangered species
Convention for the Conservation of Antarctic Marine Living Resources (CCAMLR)	Prevention of overfishing in the Antarctic and conservation of fish stocks essential to the nourishment of populations
United Nations Framework Convention on Climate Change (UNFCCC)	Dissuasion of fossil fuel usage to decrease pollution levels and increases in global warming
UN High Seas Marine Protected Areas/Areas Beyond National Jurisdiction (ABNJ)	Exploration of marine genetic resources for use in medicines
Pandemic preparedness treaty and future treaty on plastic pollution prevention	Global health security to prevent future public health emergencies of international concern (PHEIC), as well as direct effects of marine plastic pollution on human health

- The UN Convention on Biological Diversity (CBD) 30x30 targets (i.e., 30% of marine waters protected by 2030 [102]
- The World Trade Organization (WTO) Fisheries Subsidies Agreement [103]
- The UN Convention on the Law of Sea Treaty on Marine Biodiversity in Areas Beyond National Jurisdiction ([BBNJ] [104]
- The UN Sustainable Development Goals, which include SDG 14, ‘Life Below Water: Conserve and Sustainably Use Oceans, Seas and Marine Resources for Sustainable Development’ [105]
- The UN designation of 2021–2030 as the International Ocean Decade, with the strapline ‘The Science We Need for the Ocean We Want’ (https://oceandecade.org).

The next step in the evolution of these ocean policy initiatives must be the explicit inclusion in these policies of human health and human rights, key elements that at present are largely absent. If these strategies for protecting the ocean are to be effective and sustainable, they must go beyond enhancing ocean health and acknowledge the essential links between humanity and the ocean. They must advance social justice, protect human rights, especially the rights of disadvantaged people, and incorporate ‘a preferential option for the poor’. They must move ecological thinking beyond narrowly ‘green’ concerns and put people in the landscape [45,106].

**Figure 4:**
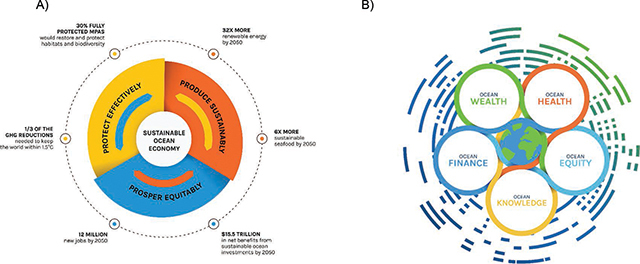
The Ocean Panel (https://oceanpanel.org/) A) A Sustainable Ocean Economy can Create a Triple Win for People, Nature and the Economy. (Originally published in: Stuchtey et al. 2020 Ocean Solutions that Benefit People, Nature and the Economy [107]); and B) the 5 Key Areas of Transformation: Health, Equity, Knowledge, Wealth, Finance (Originally published in: High Level Panel for a Sustainable Ocean Economy. 2020. Transformations for a Sustainable Ocean Economy: A Vision for Protection, Production and Prosperity. Washington, DC: World Resources Institute [13]).

To protect both ocean health and human health, 18 heads of state from diverse nations have joined together under the banner of the High Level Panel for a Sustainable Ocean Economy – the ‘Ocean Panel’ (www.oceanpanel.org). This panel’s goal is to sustainably manage 100% of the ocean area under national jurisdictions. These leaders believe that strategies for creation of a truly sustainable ocean economy must encompass effective protection, sustainable production, and equitable prosperity, and that these elements must all be aligned to create a ‘triple win’ for people, nature, and the economy (Figure 4).

To inform and advance their work, the Ocean Panel has commissioned more than 20 Blue Papers and Special Reports, created with input from over 350 experts across 54 countries (https://oceanpanel.org/publications/). These research outputs have explored myriad topics, including environmental threats to the ocean, ocean-based advances in renewable energy, coastal restoration, sustainable practices within fisheries, and marine transportation, most recently focusing on ocean and human health [108].

### Our recommendations

**Box 2. Three Key Overarching Actions for Ocean and Human Health**

- **Protect, restore, and manage ocean biodiversity**: The great potential of marine medicines and biotechnology and marine food sources depends on equitably, collaboratively, and effectively protecting and managing marine biodiversity.
*Our Recommendation* *: Ratify and implement – with genuine commitment to effective management for biodiversity protection, equity, and human wellbeing goals – the United Nations Convention on Biological Diversity (CBD) 30x30 framework, the WTO Fisheries Subsidies Agreement, and the United Nations Convention on the Law of Sea Treaty on Marine Biodiversity in Areas Beyond National Jurisdiction (BBNJ) in collaboration with local resources users.*
  These are global actions of overarching importance that can and will protect and restore the ocean, improve human health and wellbeing, and reduce stressors on ocean ecosystems. The resourced, equitable, and intentional implementation of MPAs and OECMs is a no-regret solution with clear benefits for both ocean and human health.
- **Combat climate change and eliminate pollution**: The health of coastal and island populations depends on slowing climate change to prevent extreme weather events and limit sea level rise, and limiting to the greatest degree possible all pollution from reaching the ocean. This will conserve healthy marine food sources, with particular focus on reducing emissions from fossil fuels to net zero by 2050 and eliminating plastic pollution.
*Our Recommendation* *: Uphold the commitments of countries to the UNFCCC Paris Agreement, the COP 28 outcomes, and the UN Global Plastics Treaty, currently in negotiation.*
  To be protective of human health and wellbeing in countries around the world, and especially the health of wellbeing of people in the world’s most disadvantaged nations, the UN Global Plastics Treaty must impose a mandatory cap on global plastic production, mechanisms to curb the manufacture of single-use plastics, and strict safety requirements on the more than 10,000 synthetic chemicals added to plastics. The EU Zero Pollution vision is a cross-cutting objective contributing to the UN 2030 Agenda for Sustainable Development and complementing its 2050 climate-neutrality goal. It steers the EU towards the 2050 vision of a ‘Healthy Planet for All’ by setting key 2030 targets to speed up pollution reduction.
- **Improve measurement to support equity**: It is essential to integrate measures of ocean health and human health, as well as metrics of natural capital and human capital, within ongoing monitoring, prevention, and evaluation programmes and to make data from these programmes widely available.
*Our Recommendation* *: Incorporate the evidence and linked indicators of both ocean health and human health into all policies and decision making around ocean* *–* *human interactions.*
  Through continued measurement of indicators of both human and ocean health, effectiveness can be assessed, unintended consequences detected, policies improved, and course corrections made towards long-term prevention, sustainability, and equity. These monitoring data and appropriate expertise must be shared widely, including with the Global South, and made available and accessible.

**Figure 5:**
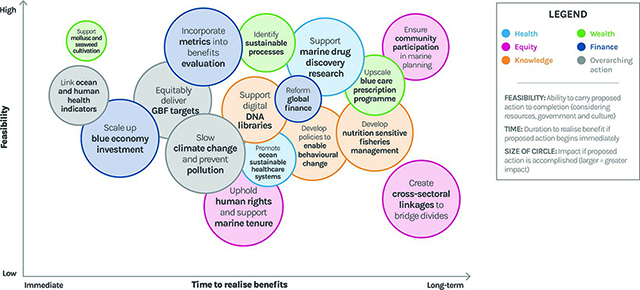
Opportunities for action to support both ocean health and human health and wellbeing in a changing planetary environment. Circles are plotted by feasibility (Y axis – the ability to carry proposed action to completion considering resources, government, and culture) and time to realise benefits (X axis – the duration required to realise benefits of proposed action). The relative size of the circle reflects the magnitude of impact of the action (in terms of overall benefit to ocean and human health globally). All actions need to be initiated immediately. Note that this figure is included primarily as a visual aid for readers. It is based on the expert opinion of authors employing best available evidence, not a quantitative analysis of all available information (Fleming, Landrigan et al. 2024 [108]).

We identify three key actions of overarching importance (Box 2):

To sustain, protect, and expand both ocean and human health, these actions must begin now. Despite positive first steps, far more needs to be done urgently. In addition to our three overarching recommendations, we offer a series of more specific recommendations in the five areas identified by the Ocean Panel: health, equity, knowledge, wealth, and finance (see Table 3 and Figure 4 and Figure 5) [13]:

**Table 3:** Tabulation of opportunities for action to support both ocean health and human health and wellbeing in a changing planetary environment. All actions need to be initiated immediately (source: Fleming, Landrigan et al. 2024 [108]).

***Overarching***	Equitably deliver the 30 x 30 target, with genuine commitment to equity and human wellbeing goals and integrating OECMs
	Slow climate change and prevent pollution from reaching the ocean, particularly by reducing greenhouse gas emissions from fossil fuels to net zero by 2050 and eliminating plastic pollution
	Link ocean and human health indicators together for ongoing monitoring, prevention, and evaluation with shared data
***Health***	Support equitable and sustainable marine medicine discovery research
	Promote ocean sustainable healthcare systems
***Equity***	Uphold human rights and support marine tenure for local communities and Indigenous Peoples
	Ensure community co-creation and involvement in marine planning
	Create cross-sectoral linkages to bridge divides and encourage co-creation
***Knowledge***	Create and support digital DNA libraries containing the genetic blueprints for most marine life
	Promote sustainable nutrition sensitive aquaculture and fisheries management
	Develop policies to enable behavioural change to foster pro-environmental behaviour
***Wealth***	Identify processes and products to be developed as socially relevant, economically sustainable, and environmentally friendly
	Support sustainable seafood (mollusc and seaweed) cultivation and harvest
	Upscale blue care prescription programmes
***Finance***	Scale up investment in a sustainable and equitable ocean economy
	Incorporate metrics of natural capital and human capital into all benefits evaluations
	Reform global finance and trade to provide more equitable access to marine resources

## Ocean and Human Health Actions

*Governments must take action to effectively conserve and manage biodiversity, encourage increased investment support for sustainable and ethical marine biotechnology start-ups with benefit sharing, and support responsible transdisciplinary research.* This must be co-designed and implemented with the participation of local communities and other potentially impacted stakeholders.

*All industries (including healthcare) must minimise their ocean footprint, helping restore what has been lost and including good ocean stewardship in sustainability strategies*. Polluters should pay, instead of profiting from the destruction of the ocean and other ecosystems. Systematic incentive structures are needed for industries to invest in long-term sustainable practices. The goal must be to create the inclusive, accessible, clean, productive, and resilient ocean called for in the UN Ocean Decade (https://oceandecade.org): an ocean that equitably and sustainably benefits the health and wellbeing of all global citizens now and in future generations.

*Collaboration between the healthcare and supplier industries (and other sectors) with improved education and communication* can lead to changes in procurement frameworks, minimising ocean impacts from product manufacture, use, disposal, and marine logistics. As an example, seaweed-derived home-compostable bioplastics can be utilised for community healthcare products, reducing the need for fossil fuel derived single-use plastics (see Figure 3).

*Researchers and public health organisations must scan the horizon for emerging climate-associated disease threats* such as antimicrobial-resistant (AMR) water- and vector-borne diseases in the ocean, which not only produce disease in individuals and communities, but collectively strain healthcare systems, making it more difficult for them to address their climate and pollution footprint.

## Ocean Equity Actions

*Ensure genuine engagement of coastal communities, small-scale fishers , and Indigenous Peoples in local marine planning, recognising traditional territories and/or incorporating OECMs*. The resilience of the coastal communities is part of the resilience assessment of any coastal area.

*Uphold human rights and support the marine tenure of local communities and Indigenous Peoples* to help support stewardship of the ocean and the security of food, livelihoods, and a way of life. This needs to be made explicit when new governance structures are introduced.

*Create new institutional structures to facilitate multi-actor, cross-sectoral collaborations.* Responsible business practices that engage and co-create with coastal communities, creating inclusive governance in planning and decision-making processes, are essential. However, we need to go further to bridge the organisational and community divides and address the challenges of the full socio-economic ecological system, and enable human and ocean health.

## Ocean Knowledge Actions

*Enhance ocean skills and knowledge by investing in DNA libraries* containing the genetic blueprints of marine life in the ocean. The rich genetic biodiversity of marine species is threatened by habitat destruction, overexploitation, land-based development and pollution, climate change, de-oxygenation, and ocean acidification. We can better appreciate, manage, and sustainably utilise the species in the ocean if this resource is jointly owned by governments to support ethical investment and private–public partnerships to develop ocean-sourced medicines and a variety of new products using the DNA blueprints.

*Develop and share technologies to produce sustainable nutrition-sensitive marine food*. The ocean provides valuable food and nutrition security to many people. Seafoods are generally good sources of dietary micronutrients (e.g., omega-3) which can be increased sustainably and equitably. However, we must address increasing inequalities in seafood distribution and consumption.

*Share scientific data and expertise* through collaborations and partnerships between global institutions and coastal communities to sustainably and equitably manage marine food production already impacted by a combination of climate change, overfishing, pollution, and processes of globalisation. For example, supporting all actors involved in small-scale fisheries, including women, who have knowledge useful for the sustainable management of extractive marine food such as seaweeds and molluscs.

*Foster pro-ocean policies that will ensure pro-environmental behaviour* through knowledge-sharing, ocean literacy and citizenship, increasing sustainable and high-quality blue space access, and appealing to the personal and societal values of the communities. This is particularly important for the healthcare sector: significantly undervalued, the ocean provides many benefits to human health and wellbeing.

## Ocean Wealth Actions

*Identify management processes and ocean products that can be developed as socially relevant, economically sustainable, and environmentally friendly* so that the ocean can continue to produce sustainably for future generations. The ocean holds important, still undiscovered, potential for new medicines, new materials and new products.

*Monitor overfishing, and stop illegal seafood harvesting*. Seafood plays an essential role in the diet of the world’s populations, providing proteins and micronutrients. But these resources must not be diverted (as they are currently through mostly legal means) to the exclusive benefit of the richest countries.

*Support mollusc and seaweed cultivation and harvest.* The development of food technologies with a low carbon footprint should be encouraged. In addition to their potential as food, seaweed has many other potential sustainable uses: animal feed, fertilisers, bioplastics, and biofuels.

*Upscale existing, and develop new, blue care prescription programmes*. The cost to society of chronic diseases (cardiovascular, mental health) has been demonstrated, as has the contribution to health and wellbeing of access to marine and other blue space areas (‘blue health effect’). Make the ocean sustainably and equitably accessible for targeted intervention programmes to reduce health-related and other costs.

## Ocean Finance Actions

*Deliver investment pathways for a blue health economy that align with Sustainable Ocean Plans* for 100% of the ocean. These would facilitate access to effective healthcare, blue foods, and clean water for all coastal and island communities, and increase opportunities to engage in marine research and product development across the globe. Marine ecosystems are underfunded and the opportunities for returns are often not fully appreciated, yet the world economy depends on a healthy blue planet.

*Reform global finance and trade to provide more equitable access to marine resources.* Build on recent multilateral successes (e.g., the new UN High Seas Treaty and the WTO Fisheries Subsidies Agreement) to phase out selected fisheries subsidies: 1) redirect finance to support investment in technologies that support sustainable production practices and efforts to meet global climate targets; 2) slow the removal of nutritious aquatic foods from generally poorer nations with high prevalence of nutritional deficiencies to higher income ones; and 3) ensure trade works for low-income nations, through debt relief and aligning trade with domestic food security policy. These efforts could target other subsidies that lead to overfishing and overcapacity but remain in place.

*Link the health and ocean finance institutional efforts with a focus on ocean-positive actions and blue infrastructure investments* that optimise the use of nature-based solutions (NBS) to deliver equitable returns to human and ocean health. By integrating ocean wellbeing solutions systemically into public health and social care, we can reduce costs and improve outcomes.

This requires key reforms of the global trade and finance system, integrating inclusive and ethical accounting for both nature and human wellbeing with involvement of affected communities. Public–private partnerships and insurance solutions can serve to deliver further support and introduce price signals. Using these formats will help to reallocate risk and encourage private sector engagement alongside public funders.

## Conclusion

We must act now and work collaboratively with individuals, communities, businesses, policymakers, the healthcare sector, academia, and governments from around the world to engage in a new vision of ocean citizenship and planetary stewardship.

Because health professionals are trusted members of societies, who are advocates for their patients and communities, the health sector is uniquely well positioned to lead the way and serve as a role model in safeguarding human health by protecting the health of the ocean [50,89,91,94].

Going forward, coastal communities, healthcare and other sectors, and many others need to join and support these global cooperative efforts for ocean and human health [109]. We support the call of the 18 heads of state who comprise the High Level Panel for a Sustainable Ocean Economy [13]:

‘We have a collective opportunity and responsibility to protect and restore the health of our ocean, and build a sustainable ocean economy that can provide food, empower coastal communities, power our cities, transport our people and goods, and provide innovative solutions to global challenges’.

Past successes combining environmental remediation with health protection and prevention give cause for hope. These victories have been enabled by international instruments and buttressed by national action. They include the global removal of lead from petrol, the Montreal Protocol ban restricting aerosol products containing chlorofluorocarbons and hydrochlorofluorocarbons, improvements in ambient air quality in a growing number of nations, and the Paris Climate Agreement. While none of these instruments is perfect, they have all achieved measurable progress, and they demonstrate that positive change is possible and attainable.

The future emphasis needs to be on applying the precautionary principle to prevent future harm, and a vision of universal ocean citizenship and planetary stewardship [25,110,111]. Only then can we provide truly transdisciplinary, science-based policy advice and action with all levels of governance, with sustainability, equity, and inclusion at the core of all their actions, to ensure that the best decisions are made for both the ocean and all people, and no one is left behind.

## References

[1] Grégoire M, Oschlies A, Canfield D, et al. Ocean Oxygen: The Role of the Ocean in the Oxygen We Breathe and the Threat of Deoxygenation; European Marine Board. 2023. https://www.marineboard.eu/sites/marineboard.eu/files/public/EMB_FSB10_Ocean_oxygen_Web-150DPI_V7.pdf.

[2] Hoegh-Guldberg O, Caldeira K, Chopin T, et al. The Ocean as a Solution to Climate Change: Updated Opportunities for Action; High Level Panel for a Sustainable Ocean Economy. 2023. https://oceanpanel.org/wp-content/uploads/2022/06/HLP_Report_Ocean_Solution_Climate_Change_final.pdf.

[3] Villasante S, Richter K, Bailey JL, et al. Building Coastal Resilience in Europe. European Marine Board; 2023. https://www.marineboard.eu/sites/marineboard.eu/files/public/publication/EMB_PP27_Coastal_Resilience_Europe_Web_v7.pdf.

[4] Falkenberg LJ, Dupont S. Climate change and the ocean. In Fleming L, Alcantara Creencia LB, Gerwick WH, et al., eds. Oceans and Human Health, Opportunities and Impacts. Academic Press; 2023;265–288. 10.1016/B978-0-323-95227-9.00025-7.

[5] Winther JG, Dai M, Rist T, et al. Integrated ocean management for a sustainable ocean economy. Nat Ecol Evol. 2020;4:1451–1458. 10.1038/s41559-020-1259-6. 32807947

[6] Madarcos JRV, Creencia LA, Roberts B, et al. Understanding Local Perceptions of the Drivers/Pressures on the Coastal Marine Environment in Palawan, Philippines. Front Mar Sci. 2021:8. https://www.frontiersin.org/articles/10.3389/fmars.2021.659699.

[7] Haque SS, Bennett BJ, Brewer TD, et al. Marine protected area expansion and country-level age-standardized adult mortality. Ecohealth. 2023;20:236–248. 10.1007/s10393-023-01658-3. 38114749 PMC10757699

[8] Nowakowski AJ, Canty SWJ, Bennett NJ, et al. Co-benefits of marine protected areas for nature and people. Nat Sustain. 2023;6(10):1210–1218. 10.1038/s41893-023-01150-4.

[9] Ban NC, Gurney GG, Marshall NA, et al. Well-being outcomes of marine protected areas. Nat Sustain. 2019;2(6):524–532. 10.1038/s41893-019-0306-2.

[10] Gollan N, Barclay K. ‘It’s not just about fish’: Assessing the social impacts of marine protected areas on the wellbeing of coastal communities in New South Wales. PLoS One. 2020;15(12):e0244605. 10.1371/journal.pone.0244605. 33378377 PMC7773243

[11] Rasheed AR. Marine protected areas and human well-being – A systematic review and recommendations. Ecosyst Serv. 2020;41:101048. 10.1016/j.ecoser.2019.101048.

[12] OECD. *The Ocean Economy in 2030*. Organisation for Economic Co-operation and Development; 2016.

[13] High Level Panel for a Sustainable Ocean Economy. Transformations for a Sustainable Ocean Economy: A Vision for Protection, Production and Prosperity. World Resources Institute; 2020. https://oceanpanel.org/the-agenda/; https://oceanpanel.org/wp-content/uploads/2022/06/transformations-sustainable-ocean-economy-eng.pdf.

[14] Antunes EM, Beukes DR, Caro-Diet EJE, et al. Medicines from the sea. In Fleming L, Alcantara Creencia LB, eds. Oceans and Human Health, Opportunities and Impacts. Academic Press; 2023.

[15] Bouley TA, Machalaba C, Keast J, et al. Marine biotechnology: A One Health approach to linking life on land to life underwater. In In Fleming L, Alcantara Creencia LB, et al., eds. Oceans and Human Health, Opportunities and Impacts. Academic Press; 2023;149–180.

[16] CHEMnetBASE. Dictionary of Natural Products. 2023. Accessed 23 October 2023. https://dnp.chemnetbase.com/chemical/ChemicalSearch.xhtml?dswid=−5745.

[17] Pascual Alonso I, Almeida García FK, Valdés Tresanco ME, et al. Marine invertebrates: A promissory still unexplored source of inhibitors of biomedically relevant metallo aminopeptidases belonging to the M1 and M17 families. Mar Drugs. 2023;21(5). 10.3390/md21050279. PMC10221039 37233473

[18] FAO Duke University & WorldFish. Illuminating Hidden Harvests – The Contributions of Small-Scale Fisheries to Sustainable Development. Food and Agriculture Organization of the United Nations; 2022.

[19] Maycock B, Then AYH, Taufek NM, et al. Food from the ocean. In Fleming L, Alcantara Creencia LB, Gerwick WH, et al., eds. Oceans and Human Health, Opportunities and Impacts. Academic Press; 2023;71–101.

[20] Tigchelaar M, Leape J, Micheli F, et al. The vital roles of blue foods in the global food system. Glob Food Secur. 2022;33:100637. 10.1016/j.gfs.2022.100637. https://www.sciencedirect.com/science/article/pii/S2211912422000281. 38285816

[21] Naylor RL, Hardy RW, Buschmann AH, et al. A 20-year retrospective review of global aquaculture. Nature. 2021;591(7851):551–563. 10.1038/s41586-021-03308-6. 33762770

[22] Golden CD, Allison EH, Cheung W. Nutrition: Fall in fish catch threatens human health. Nature. (7607): 317–320. 10.1038/534317a. 27306172

[23] White MP, Elliot LR, Gascon M, et al. Blue space, health and well-being: A narrative overview and synthesis of potential benefits. Environ Res. 2020;191:110169. 10.1016/j.envres.2020.110169. 32971082

[24] Fleming LE, Maycock B, White MP, Depledge MH. Fostering human health through ocean sustainability in the 21st century. People Nat. 2019;1(3):276–283. 10.1002/pan3.10038.

[25] Nash KL, Cvitanovic C, Fulton EA, et al. Planetary boundaries for a blue planet. Nat Ecol Evol. 2017:1(11);1625–1634. 10.1038/s41559-017-0319-z. 29066813

[26] Falkenberg LJ, Bellerby RGJ, Connell SD, et al. Ocean acidification and human health. IJERPH. 2020;17(12):4563. 10.3390/ijerph17124563. 32599924 PMC7344635

[27] Landrigan PJ, Stegeman J, Fleming LE, et al. Human health and ocean pollution. Ann Glob Health. 2020;86(1):151. 10.5334/aogh.2831. 33354517 PMC7731724

[28] Lelieveld J, Klingmüller K, Pozzeret A, et al. Effects of fossil fuel and total anthropogenic emission removal on public health and climate. Proc Natl Acad Sci U S A. 2019;116(15):7192–7197. 10.1073/pnas.1819989116. 30910976 PMC6462052

[29] Hamley GJ. The implications of seabed mining in the area for the human right to health. RECIEL. 2022;31(3):389–398. 10.1111/reel.12471.

[30] Miller KA, Brigden K, Santillo D, et al. Challenging the need for deep seabed mining from the perspective of metal demand, biodiversity, ecosystems services, and benefit sharing. Front Mar Sci. 2021:8. https://www.frontiersin.org/articles/10.3389/fmars.2021.706161.

[31] Short RE, Cox DTC, Tan YL, et al. Review of the evidence for oceans and human health relationships in Europe: A systematic map. Environ Int. 2021;146:106275. 10.1016/j.envint.2020.106275. https://www.sciencedirect.com/science/article/pii/S0160412020322303. 33242730

[32] Germond-Duret C, Heidkam CP, Morrissey J. In: Justice and the blue economy. The Geogr J. 2022;00:184–192. 10.1111/geoj.12483.

[33] Das J. Blue economy, blue growth, social equity and small-scale fisheries: A global and national level review. Soc Sci Res. 2023:4. 10.22158/sssr.v4n1p38.

[34] Reimann L, Vafeidis AT, Honsel LE. Population development as a driver of coastal risk: Current trends and future pathways. Cambridge Prisms: Coastal Futures. 2023;1:e14.

[35] FAO. The State of the World Fisheries and Aquaculture 2022. Food and Agriculture Organisation; 2022.

[36] Northrop E, Schuhmann P, Burke L, et al. Opportunities for Transforming Coastal and Marine Tourism: Towards Sustainability, Regeneration and Resilience. World Resources Institute; 2022. https://oceanpanel.org/wp-content/uploads/2022/06/Sustainable-Tourism-Full-Report.pdf.

[37] Haque N, Parveen S, Tang T, et al. Marine natural products in clinical use. Mar Drugs. 2022;20(8):528. 10.3390/md20080528. Erratum in: Mar Drugs. 2022; 20(10): PMID: ; PMCID: . 36005531 PMC9410185

[38] United Nations. Blue Economy Concept Paper. United Nations; 2014. https://sustainabledevelopment.un.org/content/documents/2978BEconcept.pdf.

[39] Cisneros-Montemayor AM, Moreno-Báez M, Reygondeau G, et al. Enabling conditions for an equitable and sustainable blue economy. Nature. 2021;591(7850):396–401. 10.1038/s41586-021-03327-3. 33731948

[40] Fleming LE, Alcantara Creencia LB, Gerwick WH, et al. Oceans and Human Health: Opportunities and Impacts. 2nd ed. Academic Press; 2023.

[41] Landrigan PJ, Raps H, Cropper M, et al. The Minderoo-Monaco Commission on plastics and human health. Ann Glob Health. 2023;89(1):23. 10.5334/aogh.4056. 36969097 PMC10038118

[42] Filbee-Dexter K, Pessarrodona A, Duarte CM, et al. Seaweed forests are carbon sinks that may help mitigate CO2 emissions: A comment on Gallagher et al. ICES J Mar Sci. 2022;80(6):1814–1819. 10.1093/icesjms/fsad107. 2023.

[43] Huang Y, Fassbender AJ, Bushinsky SM. Biogenic carbon pool production maintains the Southern Ocean carbon sink. PNAS. 2023;120(18):e2217909120. 10.1073/pnas.2217909120. 37099629 PMC10160987

[44] Newton JM, Fleming LE, Depledge MH, et al. Estimating the impact of oceans on human health: The value of taking a burden of disease approach. In: Fleming L, Alcantara Creencia LB, Gerwick WH, et al., eds. Oceans and Human Health, Opportunities and Impacts. Academic Press: 2023;473–449. 10.1016/B978-0-323-95227-9.00005-1.

[45] Whitmee S, Haines A, Beyrer C, et al. Safeguarding human health in the Anthropocene epoch: Report of The Rockefeller Foundation Commission on planetary health. The Lancet. 2015;386(10007):1973–2028. 10.1016/S0140-6736(15)60901-1. 26188744

[46] Fleming LE, Depledge M, Bouley T, et al. The ocean decade – opportunities for oceans and human health programs to contribute to public health. Am J Public Health. 2021;111(5):808–811. 10.2105/AJPH.2021.306229. 33826386 PMC8034031

[47] Franke F, Blenckner T, Duarte CM, et al. Operationalizing ocean health: Toward integrated research on ocean health and recovery to achieve ocean sustainability. One Earth. 2020;2(6):557–565. 10.1016/j.oneear.2020.05.013.

[48] WHO. Summary report on proceedings and final acts of the International Health Conference. Geneva: World Health Organization 1946.

[49] WHO. Health promotion glossary of terms. 2021. https://www.who.int/publications/i/item/9789240038349.

[50] Abbasi K, Ali P, Barbour V, et al. Time to treat the climate and nature crisis as one indivisible global health emergency. BMJ. 2023;383:2355. 10.1136/bmj.p2355. http://www.bmj.com/content/383/bmj.p2355.abstract. PMC10793827 37929357

[51] Luesch H, Moore RE, Paul VJ, et al. Isolation of dolastatin 10 from the marine cyanobacterium Symploca species VP642 and total stereochemistry and biological evaluation of its analogue symplostatin 1. J Nat Prod. 2001;64(7):907–910. 10.1021/np010049y. 11473421

[52] Vijayan SR, Prakash S, Ramasubburayan R, et al. Seaweeds: A resource for marine bionanotechnology. Enzyme Microb Technol. 2016;95:45–57. doi:10.1016/j.enzmictec.2016.06.009. https://www.sciencedirect.com/science/article/pii/S0141022916301119. 27866626

[53] Pessarrodona A, Franco-Santos RM, Wright LS, Vanderklift MA, Howard J, Pidgeon E, Wernberg T Filbee-Dexter K. Carbon sequestration and climate change mitigation using macroalgae: a state of knowledge review. Biological Reviews. 2023;98:1945–1971. 10.1111/brv.12990. 37437379

[54] Wells ML, Potin P, Craigie JS, et al. Algae as nutritional and functional food sources: Revisiting our understanding. J Appl Phycol. 2017;29(2):949–982. 10.1007/s10811-016-0974-5. 28458464 PMC5387034

[55] Nabti E, Jha B, Hartmann A. Impact of seaweeds on agricultural crop production as biofertilizer. Int J Environ Sci Te. 2017;14(5):1119–1134. 10.1007/s13762-016-1202-1.

[56] Roque BM, Venegas M, Kinley RD, et al. Red seaweed (*Asparagopsis taxiformis*) supplementation reduces enteric methane by over 80 percent in beef steers. PloS One. 2021;16(3):e0247820. 10.1371/journal.pone.0247820. 33730064 PMC7968649

[57] Hotaling S, Kelley JL, Frandsen PB. Toward a genome sequence for every animal: Where are we now? Proc Natl Acad Sci. 2021;118(52):e2109019118. 10.1073/pnas.2109019118. 34862323 PMC8719868

[58] Blasiak R, Jouffray J-P, Wabnitz CCC, Sundstrom E, Osterblom H. Corporate control and global governance of marine genetic resources. Sci Adv. 2018;4(6). 10.1126/sciadv.aar5237. PMC5990308 29881777

[59] Golden CD, Koehn JZ, Shepon A, et al. Aquatic foods to nourish nations. Nature. 2021;598(7880):315–320. 10.1038/s41586-021-03917-1. 34526720 PMC10584661

[60] Beal T, Massiot E, Arsenault JE, et al. Global trends in dietary micronutrient supplies and estimated prevalence of inadequate intakes. PLoS One. 2017;12(4):e0175554. 28399168 10.1371/journal.pone.0175554 PMC5388500

[61] Robinson JPW, Nash KL, Blanchard JL, et al. Managing fisheries for maximum nutrient yield. Fish. 2022;23(4):800–811. 10.1111/faf.12649. PMC9303942 35912069

[62] Borg B, Jouffray J-B, Wabnitz CC, et al. Randomised controlled trial to test the effectiveness of a locally-produced ready-to-use supplementary food (RUSF) in preventing growth faltering and improving micronutrient status for children under two years in Cambodia: A study protocol. Nutr J. 2018;17(1):39. 10.1186/s12937-018-0346-x. 29548287 PMC5857085

[63] Borg B, Mihrshahi S, Laillouet A, et al. Locally-procured fish is essential in school feeding programmes in sub-Saharan Africa. BMC Public Health. 2019;19(1):1200. 10.1186/s12889-019-7445-2. 31470824 PMC6717373

[64] Ahern MB, Thilsted SH, Kjellevold M, et al. Locally-procured fish is essential in school feeding programmes in sub-Saharan Africa. Foods. 2021;10(9). 10.3390/foods10092080. PMC8466277 34574190

[65] Stevens GA, Beal T, Mbuya MNN, et al. Micronutrient deficiencies among preschool-aged children and women of reproductive age worldwide: A pooled analysis of individual-level data from population-representative surveys. The Lancet Global Health. 2022;10(11):e1590–e1599. 10.1016/S2214-109X(22)00367-9. 36240826 PMC10918648

[66] Myers SS, Zanobetti A, Kloog I, et al. Increasing CO2 threatens human nutrition. Nature. 2014;510(7503):139–142. doi:10.1038/nature13179. 24805231 PMC4810679

[67] Jomaa LH, McDonnell E, Probart C. School feeding programs in developing countries: Impacts on children’s health and educational outcomes. Nutr Rev. 2011;69(2):83–98. 10.1111/j.1753-4887.2010.00369.x. 21294742

[68] Nash KL, MacNeil MA, Blanchard JL, et al. Trade and foreign fishing mediate global marine nutrient supply. Proc Natl Acad Sci. 2022a;119(22):e2120817119. 10.1073/pnas.2120817119. 35605118 PMC9295801

[69] Nash KL, van Putten I, Alexander KA, et al. Oceans and society: Feedbacks between ocean and human health. Rev Fish Biol Fish. 2022b;32(1):161–187. 10.1007/s11160-021-09669-5. 34366579 PMC8335471

[70] WHO. *Constitution of the World Health Organisation*. 2023. Accessed 23 October 2023. https://www.who.int/about/accountability/governance/constitution.

[71] Garrett JK, Clitherow TJ, White MP, et al. Coastal proximity and mental health among urban adults in England: The moderating effect of household income. Health Place. 2019;59:102200. 10.1016/j.healthplace.2019.102200. 31582294

[72] Pouso S, Borja A, Fleming LE, et al. Contact with blue-green spaces during the COVID-19 pandemic lockdown beneficial for mental health. Sci Total Environ. 2021;756:143984. 10.1016/j.scitotenv.2020.143984. 33277006 PMC7688424

[73] Maharja C, Praptiwi RA, Richter I, et al. The people of the seas and the seas of the people. In: Fleming L, Alcantara Creencia LB, Gerwick WH, et al., eds. Oceans and Human Health, Opportunities and Impacts. Academic Press; 2023a;499–530.

[74] Maharja C, Praptiwi RA, Roberts BR, et al. Sea swimming and snorkeling in tropical coastal blue spaces and mental well-being: Findings from Indonesian island communities during the COVID-19 pandemic. J Outdoor Recreat Tour. 2023b;41:100584. 10.1016/j.jort.2022.100584. https://www.sciencedirect.com/science/article/pii/S2213078022001062. 37521265 PMC9650564

[75] Richter I, Avillanosa A, Cheung V, et al. Looking through the COVID-19 window of opportunity: Future scenarios arising from the COVID-19 pandemic across five case study sites. Front Psychol. 2021:12. https://www.frontiersin.org/articles/10.3389/fpsyg.2021.635686. 10.3389/fpsyg.2021.635686 PMC8293277 34305710

[76] IHME. Global Burden of Disease (GBD). Accessed 23 October 2023. https://www.healthdata.org/research-analysis/gbd.

[77] Wildlife Trusts. A Natural Health Service: Improving Lives and Saving Money. Wildlife Trusts; 2023.

[78] Praptiwi RA, Maharja C, Fortnam M, et al. Tourism-based alternative livelihoods for small island communities transitioning towards a blue economy. Sustainability. 2021;13(12). 10.3390/su13126655.

[79] Estradivari M, Agung F, Adhuri DS, et al. Marine conservation beyond MPAs: Towards the recognition of other effective area-based conservation measures (OECMs) in Indonesia. Mar Policy. 2022;137:104939. 10.1016/j.marpol.2021.104939.

[80] Bennett NJ, Whitty TS, Finkbeineret E, et al. Environmental stewardship: A conceptual review and analytical framework. Environ Manag. 2018;61(4):597–614. 10.1007/s00267-017-0993-2. PMC5849669 29387947

[81] United Nations Environment Programme (UNEP). Inclusive Wealth Report 2023: Measuring sustainability and equity. 2023. https://wedocs.unep.org/20.500.11822/43131.

[82] Arrow K, Dasgupta P, Goulder L, et al. Are we consuming too much? J Econ Perspect. 2004;18:147–172. doi:10.1257/0895330042162377.

[83] Schindler Murray L, Milligan B, Ashford O, et al. The blue carbon handbook: Blue carbon as a naturebased solution for climate action and sustainable development. High Level Panel for a Sustainable Ocean Economy. 2023. https://oceanpanel.org/wp-content/uploads/2023/06/23_REP_HLP_Blue-Carbon-Handbook_low-res.pdf.

[84] OECD. *Social innovation*. 2023. Accessed 1 November 2023. https://www.oecd.org/regional/leed/social-innovation.htm.

[85] Merz JJ, Barnard P, Rees WE, et al. World scientists’ warning: The behavioural crisis driving ecological overshoot. Sci Prog. 2023;106(3). 10.1177/00368504231201372. PMC10515534 37728669

[86] Béné C, Hersoug B, Allison EH. Not by rent alone: Analysing the pro-poor functions of small-scale fisheries in developing countries. Dev Policy Rev. 2010;28(3):325–358. 10.1111/j.1467-7679.2010.00486.x.

[87] Islam MM, Aktar R, Nahiduzzama M, et al. Not by rent alone: Analysing the pro-poor functions of small-scale fisheries in developing countries. Sustainability. 2018;10(4). 10.3390/su10041254.

[88] Islam MM. Country Report, Bangladesh Section: Social Innovations for Transforming the Governance of Small-Scale Fisheries in the Indian Ocean Region. Social Science Research Council; 2022.

[89] Romanello M, di Napoli C, Green C, et al. The 2023 report of the Lancet Countdown on health and climate change: The imperative for a health-centred response in a world facing irreversible harms. The Lancet. 2023;402:2346. 10.1016/S0140-6736(23)01859-7. PMC7616810 37977174

[90] McKinnon S, Breakey S, Fanuele JR, et al. Roles of health professionals in addressing health consequences of climate change in interprofessional education: A scoping review. J Clim Chang Health. 2022;5:100086. 10.1016/j.joclim.2021.100086.

[91] Patel L. Our patients need us to stand up to big oil — the industry protects profits over public health. Med Page Today. 2023. Accessed 15 December 2023. https://www.medpagetoday.com/opinion/climate-checkup/107833?trw=no.

[92] Garside R, Lovell R, Husk K, et al. Nature prescribing. BMJ. 2023;383:2745. 10.1136/bmj.p2745. 38164636

[93] Britton E, Kindermann G, Domegan C, Carlin C. Blue care: A systematic review of blue space interventions for health and wellbeing. Health Promot Int. 2020;35(1):50–69. 10.1093/heapro/day103. 30561661 PMC7245048

[94] Depledge MH, White MP, Maycock B, Fleming LE. Time and tide: Our future health and wellbeing depends on the Oceans. BMJ. 2019:366:l4671. 10.1136/bmj.l4671. http://www.bmj.com/content/366/bmj.l4671.abstract. 31315830 PMC6635814

[95] American Academy of Pediatrics. Global Climate Change and Children’s Health: AAP Recommendations. Accessed 15 March 2024. https://www.aap.org/en/patient-care/climate-change/global-climate-change-and-childrens-health-aap-recommendations/.

[96] Sijm-Eeken M, Jaspers M, Peute L. Identifying environmental impact factors for sustainable healthcare: A scoping review. Int J Environ Res Public Health. 2023;20(18):6747. 10.3390/ijerph20186747. PMID: ; PMCID: . 37754607 PMC10531011

[97] Healthcare Ocean. Healthcare ocean. Accessed 23 October 2023. https://www.healthcareocean.org/.

[98] Belkhir L, Elmeligi A. Carbon footprint of the global pharmaceutical industry and relative impact of its major players. J Clean Prod. 2019;214:185–194. 10.1016/j.jclepro.2018.11.204. https://www.sciencedirect.com/science/article/pii/S0959652618336084.

[99] Steenmeijer MA, Rodrigues JFD, Zijp MC, Waaijers-van der Loop SL. The environmental impact of the Dutch health-care sector beyond climate change: An input-output analysis. The Lancet Planetary Health. 2022;6(12):e949–e957. 10.1016/S2542-5196(22)00244-3. 36495889

[100] Li Y, Jia P, Jianget S, et al. The climate impact of high seas shipping. Natl Sci Rev. 2022; 10(3):nwac279. 10.1093/nsr/nwac279. PMID: ; PMCID: . 36875783 PMC9976761

[101] Carvalho M, Mathis J, Hamzah BA, et al. Ocean law, policies, and regulation. In: Fleming L, Alcantara Creencia LB, Gerwick WH, et al., eds. Oceans and Human Health, Opportunities and Impacts. Academic Press; 2023;643–685. 10.1016/B978-0-323-95227-9.00023-3.

[102] CBD (Conference on Biodiversity). United Nations, ed. Protected areas and other effective area-based conservation measures. Edited by United Nations, 2018. https://www.cbd.int/doc/c/0165/9fc3/962fae6c8e6d0f8bc8ca361d/mcb-em-2018-01-inf-05-en.pdf.

[103] WTO. Agreement on Fisheries Subsidies. 2022. https://www.wto.org/english/tratop_e/rulesneg_e/fish_e/fish_e.htm.

[104] UN. BBNJ Agreement under the United Nations Convention on the Law of the Sea on the conservation and sustainable use of marine biological diversity of areas beyond national jurisdiction. 2023. https://www.un.org/bbnjagreement/en.

[105] Cicin-Sain B. Goal 14 – conserve and sustainably use oceans, seas and marine resources for sustainable development. UN Chronical. Accessed 28 December 2023. https://www.un.org/en/chronicle/article/goal-14-conserve-and-sustainably-use-oceans-seas-and-marine-resources-sustainable-development.

[106] Pope Francis. Laudato Si. Encyclical Letter on Care for our Common Home. The Vatican; 2015.

[107] Stuchtey M, Vincent A, Merkl A, et al. Ocean solutions that benefit people, nature and the economy. World Resources Institute; 2020. https://oceanpanel.org/wp-content/uploads/2022/06/full-report-ocean-solutions-eng.pdf.

[108] Fleming LE, Landrigan P, Gerwick WH, et al. How can a healthy ocean improve human health and enhance wellbeing on a rapidly changing planet? Blue Paper. World Resources Institute. 2024. https://oceanpanel.org/publication/ocean-human-health/.

[109] US Ocean Policy Committee. Ocean justice strategy. The White House; 2023. https://www.whitehouse.gov/wp-content/uploads/2023/12/Ocean-Justice-Strategy.pdf?cb=1701982354.

[110] Kelly MR, Kasinak J-M, McKinley E, et al. Conceptualizing the construct of ocean identity. Ocean Sustainability. 2023;2(1):17. 10.1038/s44183-023-00025-7.

[111] O’Halloran C, Buchan PM, Bridge NL, et al. Oceans and human health stewardship, literacy, and citizenship. In: Fleming L, Alcantara Creencia LB, Gerwick WH, et al., eds. Oceans and Human Health, Opportunities and Impacts. Academic Press; 2023;745–774. 10.1016/B978-0-323-95227-9.00004-X.

